# Comparison of bacterial microleakage of three bioactive endodontic sealers in simulated underwater diving and aviation conditions

**DOI:** 10.1186/s12903-021-01699-6

**Published:** 2021-07-15

**Authors:** Mehdi Dastorani, Behnam Malekpour, Mohsen AminSobhani, Mohammadsadegh Alemrajabi, Arezoo Mahdian, Behrooz Malekpour

**Affiliations:** 1 Department of Endodontics, School of Dentistry, AJA University of Medical Sciences, End of 13th East St., Ajodanieh, Tehran, Iran; 2 School of Dentistry, AJA University of Medical Sciences, End of 13th East St., Ajodanieh, Tehran, Iran; 3 Department of Oral Medicine, School of Dentistry, AJA University of Medical Science, End of 13th East St., Ajodanieh, Tehran, Iran; 4 Orthodontic Research Center, School of Dentistry, Shiraz University of Medical Sciences, Qom Abad, Ghasrodasht St., 713451836 Shiraz, Iran; 5 Department of Prosthodontics, Isfahan (Khorasgan) Branch, Isfahan Azad University, University Blvd, Arqavanieh, Jey Street, P.O.Box: 81595-158, Isfahan, Iran

**Keywords:** Atmospheric pressure, Dental leakage, Mineral trioxide aggregate, Root canal obturation

## Abstract

**Background:**

Bacterial microleakage is an important cause of apical periodontitis and endodontic treatment failure. This study aimed to assess the bacterial microleakage of nano-mineral trioxide aggregate (nano-MTA) as a sealer, Endoseal MTA, and GuttaFlow Bioseal sealers in atmospheric pressure, and simulated underwater diving and aviation conditions.

**Methods:**

In this in vitro, experimental study, 180 extracted single-rooted teeth were cleaned and shaped, and were then randomly divided into three groups for single-cone obturation using Endoseal MTA, GuttaFlow Bioseal, or nano-MTA as a sealer. Each group was then randomly divided into three subgroups, and subjected to ambient atmospheric pressure, 2 atm pressure (to simulate underwater diving), and 0.5 atm pressure (to simulate aviation) using a custom-made pressure chamber. The teeth then underwent microbial leakage test using Streptococcus mutans (S. mutans), and the percentage of samples showing microleakage was recorded for up to 1 month, and analyzed using the Chi-square test.

**Results:**

The three sealer groups were significantly different regarding bacterial microleakage (*P* < 0.05). The nano-MTA group showed significantly higher microleakage after 15 days than the other two groups (*P* = 0.006). The effect of pressure on bacterial microleakage was not significant in any sealer group (*P* > 0.05).

**Conclusion:**

Within the limitations of this in vitro study, it may be concluded that single-cone obturation technique using nano-MTA as a sealer results in lower resistance to bacterial microleakage compared with the use of GuttaFlow Bioseal, and Endoseal MTA. Pressure changes in simulated underwater diving and aviation conditions had no significant effect on bacterial microleakage.

*Trial Registration Number* This is not a human subject research.

## Background

Apical periodontitis is a common complication caused by the activity of pathogenic microorganisms in the root canal system [1, 2]. Prevention or treatment of apical periodontitis requires chemomechanical cleaning and shaping of the root canal system, and subsequent sealing of the access cavity and root canal space [3, 4]. Success of endodontic treatment depends on creation and preservation of hermetic seal of the root canal system, which requires the use of intracanal sealers capable of well adhering to the root canal walls and the root filling materials [5].

Despite the significance of cleaning and shaping of the root canal system [6, 7], the root canal obturation materials and techniques can greatly affect the long-term success of root canal treatment [8, 9]. Although no obturation technique can provide a hermetic seal and prevent bacterial leakage by 100%, cold lateral compaction technique with the use of gutta-percha is still among the most commonly used obturation techniques [10]. In the recent years, however, the single-cone technique with the use of gutta-percha has gained increasing popularity. In this technique, the root canal is filled with one single gutta-percha cone matching the size of the largest rotary file used for root canal preparation [10].

All endodontic materials and techniques aim to minimize the access of bacteria to the periapical region. The microleakage test is a well-accepted technique for assessment of the success of endodontic materials and techniques, which determines the resistance of root filling materials to bacterial leakage [11].

Despite the numerous advantages of gutta-percha, it cannot chemically adhere to the root canal walls, and allows the leakage of bacteria through the interface of root filling material and canal wall, which is a major drawback of endodontic treatment with gutta-percha [12]. Thus, endodontic sealers are used to fill the gap between the gutta-percha and root canal walls. However, void formation in the sealer may allow the leakage of bacteria and their byproducts, and compromise the success of treatment [13–15]. Thus, type of endodontic sealer is an important factor in occurrence of bacterial leakage. Different endodontic sealers are available in the market which can be categorized into six groups of resin-based sealers, zinc oxide eugenol, calcium hydroxide, calcium silicate, glass ionomer, and silicon sealers [16].

Endoseal MTA (Maruchi, Wonju, South Korea) is a new calcium silicate sealer with many ideal properties such as optimal biocompatibility and favorable physical properties [17, 18].

GuttaFlow Bioseal (Coltene/Whaledent AG, Altstatten, Switzerland) is a silicon-based sealer containing polydimethylsiloxane, and a mixture of gutta-percha and calcium silicate particles [19]. The manufacturer of this sealer claims that it has excellent adaptation due to its optimal flowability and can undergo gradual volumetric expansion. It can well adapt to the root canal walls and can be used as a sealer or even a root filling material [20].

Nano-mineral trioxide aggregate (nano-MTA, patent application #13/211.880) is a calcium silicate-based sealer containing 40–100 nm MTA particles, which increase the active surface area for reaction. The nano-MTA particles improve the cement properties by increasing the alkalinity, enhancing the release of calcium ions, improving the hydration phase of cement, and increasing the integrity of cement by improving the interlocking of particles [21].

By an increase in the number of underwater divers and aviation personnel, dental clinicians increasingly encounter the dental effects of pressure change, such as toothache caused by a change in ambient pressure, referred to as barodontalgia, which need to be addressed [22]. Pressure change can affect the tooth structure and restorations [23]. However, the effects of pressure change on endodontically treated teeth and their bacterial leakage have not been well investigated. Moreover, it is important to find out which type of bioceramic sealer can minimize bacterial leakage under different atmospheric conditions. Thus, this study aimed to assess the bacterial microleakage of nano-MTA as a sealer, Endoseal MTA, and GuttaFlow Bioseal sealers in atmospheric pressure, and simulated underwater diving and aviation conditions.

## Methods

This in vitro, experimental study evaluated 180 single-rooted human teeth that had been extracted within the past 6 months due to hopeless periodontal prognosis or as part of orthodontic treatment. The study protocol was approved by the ethics committee of AJA University of Medical Sciences (ethical approval code: 97,000,668). The study was performed in line with the principles of the Declaration of Helsinki. All methods were performed in accordance with the relevant guidelines and regulations of AJA University of Medical Sciences.

The inclusion criteria were single-rooted, single-canal human teeth extracted within the past 6 months with no internal or external root resorption, dilaceration, severe root curvature, root caries, or previous endodontic treatment, which were assessed by periapical radiography. The teeth were selected using convenience sampling.

The sample size was calculated to be 180 teeth assuming the effect size of 0.3, 80% study power, and alpha = 0.05.

The teeth were immersed in 0.2% thymol solution for disinfection, and were then transferred to saline. The teeth were then immersed in 5.25% sodium hypochlorite (Chloraxid, Cerkamed, Poland) for 15 min to eliminate the organic debris. The residual tissues were removed by a curette.

### Access cavity preparation

Standard access cavity was prepared in all teeth. A reference point was selected on the tooth crown, and a #10 K-file (Mani, Nakanishi Inc., Tokyo, Japan) was introduced into the root canal such that the file tip was visible at the apex at × 10 magnification. Next, 16 mm was subtracted from this length, and the teeth were decoronated at the marked level by a diamond bur (Teeskavan, Iran) and high-speed handpiece under water spray. By doing so, the root canal length was standardized in all teeth (16 mm). One millimeter was subtracted from the 16 mm length to have 15 mm working length in all teeth.

### Root canal instrumentation

The root canals were instrumented by hand files (Mani, Nakanishi Inc., Tokyo, Japan) and ProTaper rotary files (Dentsply Maillefer, Ballaigues, Switzerland) with an electric endodontic motor (Endo Mate DT, NSK, Japan) operating at 250 rpm and 3 N/cm torque. First, the root canals were instrumented with hand files up to #25 K-file to the working length, and then SX ProTaper rotary file was used for coronal root canal flaring followed by S1, S2, F1, F2 and F3 files to the working length. After using each file, a #10 K-file was passed through the apex to ensure patency. Each rotary file was used for preparation of 3 teeth. Passive root canal irrigation between files was performed with 10 mL of 5.25% sodium hypochlorite solution (Chloraxid, Cerkamed, Poland) using a plastic syringe with a 27-gauge needle. After completion of root canal cleaning, the root canals were rinsed with 5 mL of 17% EDTA for 1 min (Asia Chemi Teb Mfg, Tehran, Iran) [24] followed by a final rinse with 2 mL of distilled water.

### Root canal obturation

After cleaning and shaping, the root canals were dried with paper points (Dentsply Maillefer, Ballaigues, Switzerland). Next, the teeth were randomly divided into three groups for obturation using three different sealers (n = 54). Endoseal MTA (Maruchi, Wonju, South Korea) was used in group A, nano-MTA (patent application #13/211.880) was used in group B, and GuttaFlow Bioseal (Coltene/Whaledent AG, Altstatten, Switzerland) was used in group C. Nine teeth were considered as the positive control, and 9 other teeth were considered as the negative control group.

In Endoseal MTA and GuttaFlow Bioseal groups, the sealer was passively injected into the canal by a syringe, and then a size 30 Lentulo spiral was used to spread the sealer to the working length. In the nano-MTA group, nano-MTA was prepared in paste-like consistency on a glass slab and delivered into the canal by a Lentulo spiral. The largest gutta-percha (Aurum Pro; Meta biomed, Korea) with 6% taper that reached the working length and had tug-back was used for single-cone obturation of the root canals. The gutta-percha was dipped in sealer and inserted into the canal to the working length. It was then cut 1 mm below the orifice using a heat carrier and condensed by a plugger. In the positive control group, cleaning and shaping were performed as explained earlier, but the root canals were not filled. In the negative control group, cleaning and shaping and obturation of root canals (3 teeth from each sealer group) were performed as explained earlier, and then the access cavity and apical foramen were sealed with sticky wax.

### Pressure cycles

After root canal obturation, the teeth were stored in an incubator with 95% humidity at 37 °C for 1 week. Next, the teeth in each group were randomly divided into three subgroups (n = 18) and subjected to ambient atmospheric pressure (1 atm), 2 atm pressure to simulate underwater diving, and 0.5 atm pressure to simulate aviation [23, 25]. For this purpose, the teeth were subjected to the abovementioned pressures for 45 min within every 24 h (as explained below), and were then placed back in ambient atmospheric pressure. This cycle was repeated for 1 month for each group.

### Simulation of underwater diving and aviation conditions

**Fig. 1:**
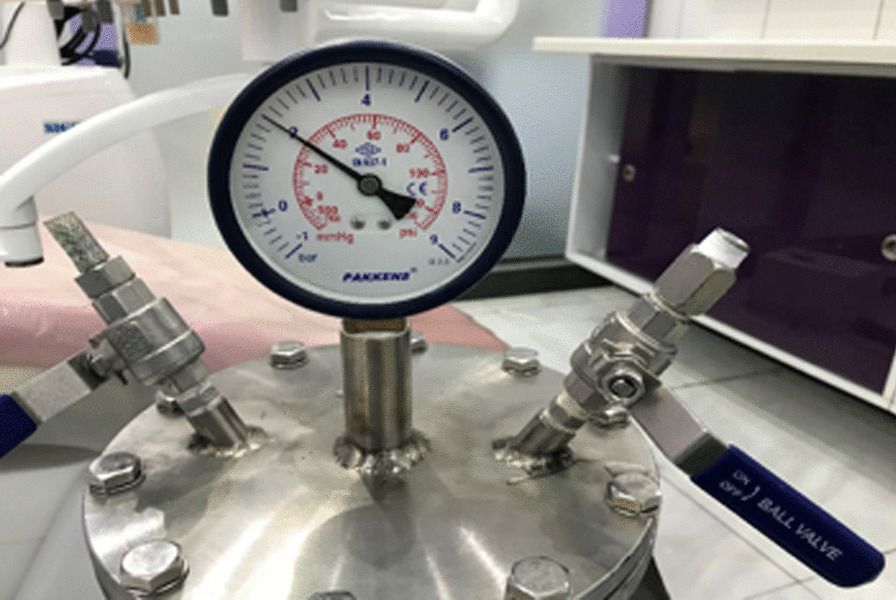
Custom-made chamber to simulate underwater diving and aviation conditions

For this purpose, a custom-made chamber with a monometer (an external pressure gauge) was experimentally designed. The chamber was pressurized with air by using a compressor, and an air-vacuum pump was used to decrease the pressure. The speed of pressure change was 1 atm/min. The pressure inside the chamber was controlled by a barometer. If the pressure was too high or low, the chamber would be depressurized or pressurized using the output and input valves, respectively (Fig. 1).

Prior to the placement of the teeth in the chambers, they were wrapped in a moist gauze and were then placed in the chamber. Increasing the pressure to 2 atm simulated 10 m depth underwater, and 0.5 atm pressure simulated the pressure at 5.5 km height from the sea level [23]. Thus, diving descent was simulated by increasing the pressure to 2 atm similar to depth of 10 m under water. Decreasing the pressure to 0.5 bars-like at 5.5 km from the sea level altitude- simulated flight ascents [23].

The control teeth, and other teeth (when not in the chamber) were stored in an incubator (Memment INC10, Germany) at 37 °C and 97% relative humidity during this time period.

### Sealing of root surfaces

The entire root surface was sealed with two layers of nail varnish except for the apical 2 mm and apical foramen, which remained exposed. This was done to prevent bacterial leakage through the accessory canals. In the positive control group, root surface was sealed as in the experimental groups while in the negative control group, the entire root surface and the apical foramen were coated with 2 layers of nail varnish.

### Bacterial leakage model

A bacterial leakage model consisting of an upper and a lower chamber was used for assessment of bacterial microleakage using Streptococcus mutans (S. mutans). The upper chamber included a 15-mm polycarbonate centrifuge tube with a small hole at one end. S. mutans in Wilkins-Chalgren culture medium was placed in this chamber and in contact with the coronal part of the roots. The teeth were placed in the tube from the apical end, and gently pressed until half of the root was inside the tube. It was then sealed with sticky wax; 4 mm of the root was placed in the upper chamber.

The lower chamber included a 15-mL tube, and the apical part of the root was placed in it. This chamber contained culture medium and phenol red indicator with 1% sucrose.

### Sterilization of specimens

The culture media were autoclave-sterilized at 121 °C for 20 min. After placement of the teeth in the chambers, they were sterilized with ethylene oxide for 12 h (Andersen sterilizer, Haw River, North California, 27,258–9564. USA). To ensure sterility, all specimens were incubated at 37 °C for 3 days. During this time period, none of the samples showed any evidence of bacterial contamination.

### Bacterial inoculation

Standard strain S. mutans (ATCC 35,668) was obtained in lyophilized form from the Iranian Research Organization for Science and Technology, and cultured in 10 cc of Wilkins-Chalgren broth for 24 h. Every 24 h, 0.2 mL of the bacterial suspension was added to the upper chamber. Every 2 days, 9 cc of Wilkins-Chalgren broth was removed from the upper chamber and replaced with fresh broth. The valve of the centrifuge tube was used to prevent evaporation and contamination of medium.

### Microleakage test

The teeth were incubated at 37 °C for 30 days, and the change in the color of pH indicator was evaluated every 5 days. Color change from red to yellow was considered as a positive result (acid production). To confirm the presence of bacterial colonies, blood agar culture medium was used. The purity of proliferated bacteria was evaluated by Gram-staining (observation of Gram-positive chains of cocci) and the ability to proliferate on selective S. mutans culture medium i.e. mitis salivarius agar.

### Statistical analysis

The mean and standard deviation of the data were reported, and comparisons were performed using the Chi-square test via SPSS version 16 at 0.05 level of significance.

## Results

All positive control teeth showed bacterial microleakage while none of the negative control teeth showed microleakage.

Table 1 presents the percentage of teeth with bacterial leakage in the three sealer groups, irrespective of pressure conditions, at different time points. As shown, at 30 days, maximum microleakage was noted in nano-MTA group (90% of the samples) while minimum microleakage was noted in Endoseal MTA group (56.5% of the samples) (*P* < 0.001). Pairwise comparisons revealed that Endoseal MTA and GuttaFlow Bioseal groups were not significantly different regarding microleakage (*P* > 0.05). Nano-MTA group had significantly higher microleakage than the other two groups (*P* < 0.05).

**Table 1:** Percentage of teeth with bacterial leakage in the three sealer groups, irrespective of pressure conditions, at different time points

Day	5	10	15	20	25	30
*Group*						
GuttaFlow Bioseal	3.7%	5.5%	22.4%	38.7%	55.2%	66.2%
Nano MTA as sealer	7.4%	11.1%	44.4%	64.75%	70.25%	90%
Endoseal MTA	5.55%	13.3%	26.7%	39.8%	51%	56.5%
*P* value	0.12	0.15	0.006	< 0.001	< 0.001	< 0.001

Table 2 shows bacterial microleakage of the subgroups at different pressure conditions. The results showed no significant difference among the three subgroups of each sealer group regarding microleakage (*P* > 0.05).

**Table 2:** Bacterial microleakage of the subgroups at different pressure conditions

Group	Subgroup	Percentage of leakage (%)	*P* value
GuttaFlow Bioseal	Ambient	60.6	0.21
	Aviation	61.0	
	Underwater diving	75.0	
Nano MTA as sealer	Ambient	80.0	0.14
	Aviation	90.0	
	Underwater diving	95.0	
Endoseal MTA	Ambient	60.0	0.34
	Aviation	58.0	
	Underwater diving	53.0	

## Discussion

This study assessed the bacterial microleakage of nano-MTA as a sealer, Endoseal MTA and GuttaFlow Bioseal sealers in atmospheric pressure, and simulated underwater diving and aviation conditions. This study was among the first to address this topic and also to find out whether specific measures need to be taken regarding the choice of sealer in endodontic treatment of underwater divers or aviators. A bacterial leakage model was used in this study to assess the quality of root canal treatment with the use of three different sealers because this model is widely used for this purpose and provides accurate, reproducible data [26]. The bacterial leakage model is often preferred to the dye penetration technique because the former better simulates the clinical setting [27]. However, it should be noted that bacterial leakage tests have some limitations in assessment of sealability of materials with antibacterial properties such as Endoseal MTA and nano-MTA. A custom-made chamber with a monometer (an external pressure gauge) was experimentally designed in this study to simulate underwater and aviation conditions according to studies by Shafigh et al., [23] and Safaie et al. [25].

The current results showed no significant difference in bacterial microleakage of the three subgroups subjected to different pressure conditions. This finding was in line with the results of Shafigh et al., [23] who evaluated the effect of pressure change on microleakage of composite restorations and showed that pressure change had no significant effect on microleakage of composite restorations. Also, Safaie et al. [25] indicated that increasing the atmospheric pressure slightly, but not significantly, increased the leakage of dye through the root canal system, which was in agreement with our results; although their method of assessment of microleakage was different from ours.

Boyle's law states that at a constant temperature, the volume of a given mass of a dry gas is inversely proportional to its pressure. The volume of voids and air bubbles trapped in restorations and filling materials changes following alterations in atmospheric pressure. For instance, in underwater diving or aviation, the atmospheric pressure decreases and subsequently, the air volume increases, which can damage the tooth structure or restorative materials [28]. Thus, it may be concluded that by minimizing void formation in restorations and root canal fillings, the adverse effects of pressure change on the root structure, restorations, and root fillings can be prevented or minimized [29]. Therefore, standard high-quality treatment can prevent or minimize the adverse effects of pressure change on the teeth. Our results confirmed this statement. This result in our study can be due to standard controlled in vitro conditions, use of single-canal teeth with no complexity or root curvature, and single-cone obturation of root canals, which simplifies root canal treatment. All these factors are responsible for simplified in vitro conditions, compared with the clinical setting, and easier prevention of void formation in vitro. Also, it should be noted that in this study, the teeth were subjected to altered pressure conditions for 45 min/day for one month. Different results may be obtained in longer periods of exposure to pressure change, which needs to be investigated in future studies. Moreover, as mentioned earlier, single-cone obturation technique plays an important role in achieving an ideal seal. This technique has many advantages; however, it may provide poor adaptation to canal walls in root canals with irregular and complex shapes. Thus, selection of this technique of obturation should be done with caution [13, 30].

Despite the availability of many sealer types, no sealer currently has ideal sealability in the long-term [31]. The current results showed significant superiority of Endoseal MTA and GuttaFlow Bioseal compared with nano-MTA for prevention of bacterial leakage. To the best of the authors’ knowledge, no previous study has compared the sealability of the abovementioned three sealers, and this study is the first to address this topic. However, Hwang et al. [31] compared microbial leakage of Endoseal MTA and GuttaFlow and reported the superiority of Endoseal MTA, which was in line with our results. They attributed this difference to chemical and mechanical properties of sealers. Dastorani et al. [32] compared the bacterial microleakage of Endoseal MTA and Pro-Root MTA in root perforation repair using a double chamber system. They reported that none of the Endoseal MTA samples showed any microleakage during the 35-day study period. They attributed the high sealing ability of Endoseal MTA to its easier application and lower technical sensitivity. These results confirmed the results of our study. Although they used the same methodology for evaluation of bacterial microleakage as ours, the bacteria used in their study were different from those in our study. Microleakage of sealers depends on void formation in the sealer, its flow, and size of sealer particles [33]. Fillers with smaller particles have higher flowability and subsequently lower leakage [25]. In the present study, nano-MTA as a sealer had lower resistance to microbial leakage despite the fact that it has the smallest size of particles. This finding may be due to the clinical properties of this sealer. Endoseal MTA is available in premixed injectable form. GuttaFlow Bioseal is a mixture of two pastes that are mixed in a syringe prior to use, which enhances its clinical application and ensures optimal integrity of the final sealer with minimal porosities. However, nano-MTA is supplied in the form of powder and liquid, which should be manually mixed. Also, MTA cement becomes highly porous after mixing, which can enhance microbial leakage [33]. According to the current results, use of nano-sized particles cannot overcome the porosity and void formation in MTA to serve as an optimal sealer. Endoseal MTA has optimal flowability and slight setting expansion, which are advantageous for reduction of microleakage [18]. Also, it is available in premixed injectable form, which decreases void formation during its application. However, Moazami et al. [34] reported that this sealer had more favorable performance when used with gutta-percha cone-mediated ultrasonic activation. Thus, they suggested gentle ultrasonic activation in clinical application of Endoseal MTA to obtain more favorable results. Another study reported that Endoseal MTA had the best adhesion to dentin and optimal wettability when compared with EndoSequence BC sealer, MTA Fillapex and AH Plus [35]. Similarly, GuttaFlow has some degrees of setting expansion as well, which is favorable [20].

Since an optimal seal can guarantee the long-term success of root canal treatments [31], microleakage should be assessed over long periods of time to obtain more reliable results.

Use of microbial leakage model, and evaluation of sealability of three commonly used sealers for the first time were among the strengths of this study. However, in vitro design, short duration of exposure to pressure change, and use of only one type of microorganism (considering the poly-microbial nature of endodontic infections) were the main limitations of this study. Also, nano-MTA and Endoseal MTA have antibacterial properties, which could have affected the results of bacterial leakage test.

Future studies with longer periods of exposure to pressure change and longer follow-ups, and on different microorganisms are required employing other microleakage assessment techniques.

## Conclusion

Within the limitations of this study, it may be concluded that single-cone root canal obturation by using nano-MTA as a sealer results in lower resistance to bacterial leakage compared with the use of GuttaFlow Bioseal, and Endoseal MTA. Pressure changes in simulated underwater diving and aviation conditions had no significant effect on bacterial microleakage.

## Data Availability

All data generated or analysed during this study are included in this published article.

## Abbreviation

MTA: Mineral trioxide aggregate
